# CEdRIC: Strategy for Patient Education During COVID-19 Triage

**DOI:** 10.5811/westjem.2020.7.47907

**Published:** 2020-10-06

**Authors:** Benoit Pétré, Jean-Christophe Servotte, Justine Piazza, Alexandre Ghuysen, Aurore Margat, Remi Gagnayre, Dieudonné Leclercq

**Affiliations:** * University of Liège, Department of Public Health, Wallonia, Belgium; † University Hospital Centre of Liège, Department of Emergency Medicine, Wallonia, Belgium; ‡ University of Sorbonne Paris Nord, Education and Health Practices Laboratory UR3412, Sorbonne Paris Cité, France; § University of Liège, Department of Education and Training, Wallonia, Belgium; ¶ Member of Be.Hive, Interdisciplinarity Primary Care Chair

## Abstract

The current coronavirus disease 2019 (COVID-19) pandemic is forcing healthcare systems around the word to organise care differently than before. Prompt detection and effective triage and isolation of potentially infected and infectious patients are essential to preventing unnecessary community exposure. Since there are as yet no medications to treat or vaccines to prevent COVID-19, prevention focuses on self-management strategies, creating patient education challenges for physicians doing triage and testing. This article describes a five-step process for effectively educating, at discharge, patients who are suspected of being infectious and instructed to self-isolate at home. We are proposing the CEdRIC strategy as a practical, straightforward protocol that meets patient education and health psychology science requirements. The main goal of the CEdRIC process is to give patients self-management strategies aimed at preventing complications and disease transmission. The COVID-19 pandemic is challenging clinicians to rapidly teach their patients self-management strategies while managing the inherent pressures of this emergency situation. The CEdRIC strategy is designed to deliver key information to patients and standardize the discharge process. CEdRIC is currently being tested at triage centres in Belgium. Formal assessment of its implementation is still needed.

## INTRODUCTION

Countries all over the world are facing a major public health security crisis related to the management of the coronavirus disease 2019 (COVID-19) pandemic. Every country will be affected, and governments around the world need to prepare a strategic response in order to minimize the impact of the disease and its spread on the morbidity and mortality of their populations, as well as the resulting social, economic, and political disruptions. A key ingredient of a healthcare system’s response to COVID-19 is the ability to institute prompt detection and effective triage and isolation of potentially infected and infectious patients, with the goal of preventing unnecessary community exposure.^1^,^2^

The vast majority of suspected COVID-19 patients experience only mild symptoms,^3^ and will be instructed to self-isolate at home while awaiting their test results. (This was the case with 77% of the patients who presented at the University Hospital of Liège triage centre from 2 March–4 May 2020.) Patients who test positive are advised to stay at home, provided they are not experiencing complications. Even those who test negative must be warned that they remain at risk of the disease. Hence, sustainability and preventing healthcare system overload will depend on people’s ability to care for themselves at home, while minimizing the risk of infecting their families.

Since there are as yet no medications to treat or vaccines to prevent COVID-19,^4^ prevention focuses on self-management strategies: symptom monitoring; appropriate and frequent hand hygiene; cough etiquette; social distancing; and strict self-isolation.^5^ Behavioural science must, therefore, be at the heart of the public health response,^6^ especially when it comes to patient education. In emergency departments (ED), in particular, recommendations enhance the standard infection prevention and control practices.^7^

**Figure 1:**
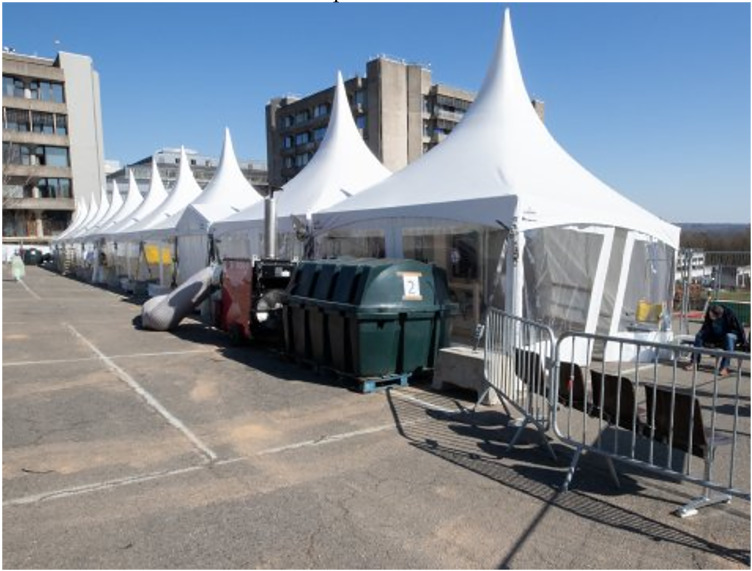
COVID triage centre, University Hospital, Liège (Belgium).

In most countries, the screening and triage of COVID-19 suspects are centralised at “triage settings.” In Belgium, triage centres have been created specifically to screen patients referred by a physician and suspected of having COVID-19. Triage and screening centres have been set up at primary care facilities: first, near hospitals, to take advantage of their resources and experienced emergency staff; and second, at other, non-primary care facilities. Triage and screening tents (Figure 1) have been erected outside those facilities to reduce the risk to other patients and staff.

These settings serve two essential functions during the pandemic: 1) Triage: examining patients sent by outside doctors and likely to be infected with COVID-19. This prevents these patients from having to go to a general practitioner’s waiting room or to a hospital ED, where they might infect others. If appropriate, they are referred to the hospital for admission. 2) Screening: testing to see whether patients are infected or not.

Patient screening and triage is a key opportunity for educating COVID-19 patients to prevent them from transmitting the disease. Effective triage should include patient education at discharge.^8^ Despite the constraints (unpredictable workload, in particular), triage and testing settings should be viewed as a good place to improve future patient adherence to recommendations, thereby preventing complications^9^ and, in this context, disease transmission. Patient education should also help health professionals (general practitioners in most cases) who receive calls from patients and arrange for remote triage. Unless there is a clinical need for in-person care, patients should be able to get advice and care without visiting the practice. Moreover, informing patients that they have COVID-19 is giving them bad news; delivering that bad news and offering education is challenging in an ED context because the patient is meeting the physician for the first time. Because – as has been previously demonstrated^10^ – clinicians lack the skills needed for this, a support tool seems important.

Although patient education is a key component in the fight against COVID-19, health providers have no clear guidance on how to proceed. Here we propose a protocol for providing basic in-person and remote patient education to suspected or confirmed cases in patients who are instructed to self-isolate at home. Patients who are admitted to hospital require special attention and are excluded from the discussion.

### The Five-step CEdRIC Strategy

While the need for patients to understand discharge instructions is well established in the literature,^11^ in emergency situations – especially mass casualty events – discharge communications may be reduced to a brief exchange,^11^ leaving patients uncertain about what to do when they return home; this is especially true for patients with low health literacy. The CEdRIC strategy is a practical, straightforward protocol that meets the requirements for effective discharge patient education adapted to the special conditions made necessary by the current situation. The CEdRIC protocol consists of five steps that clinicians can use to develop a structured approach to discharge instruction (see Table 1 for an overview of the protocol). Each step is supported by references to the education and health psychology literature.

#### Step 1 – Ensure that the patient **C**omprehends and accepts the situation

The first step after testing and triage involves giving the patient information about his condition, its potential course, and how to self-isolate at home. This information can cause great anxiety when people do not understand why they are being advised to go home while potentially infected with COVID-19. As anxiety impairs patients’ ability to take in and process new information,^12^ it is important that clinicians listen to and reassure their patients. Clinicians can use open-ended questions to determine how well the patient understands his medical situation.^13^

Jay (1996)^14^ showed that methods such as “touch, company and information” are effective in reducing anxiety in seriously injured patients. Information is the only one of these three types of action that is appropriate and applicable in triage settings. Informing patients and raising their awareness of their clinical situation involves two tasks: dealing with their emotional response; and developing a strong rationale. Dealing with a patient’s emotional response is difficult. Health professionals must strike a delicate balance between reassuring patients that it is safe to return home and convincing them of the seriousness of the situation, so that they do not minimise the problem.^15^

The large majority of patients who are at low risk should be told that in most people the disease is not as severe as the media reports, and that there are strategies for avoiding transmission to their families (see Step 2). Indeed, recent research suggests that the real-world mortality rate may be lower than previously reported and that the vast majority of suspected COVID-19 cases experience none or only mild symptoms.^3^,^16^,^17^,^18^ This could be due to the “iceberg” effect, in which there are many more patients below the surface who act as a reservoir of “spreaders” transmitting the disease to the rest of the population, and include the more vulnerable of those at risk of severe disease. Patients should, however, be warned that this new virus appears to be highly contagious,^19^ and requires strict self-isolation.

#### Step 2 –Educate the patient about self-management strategies

**Figure 2:**
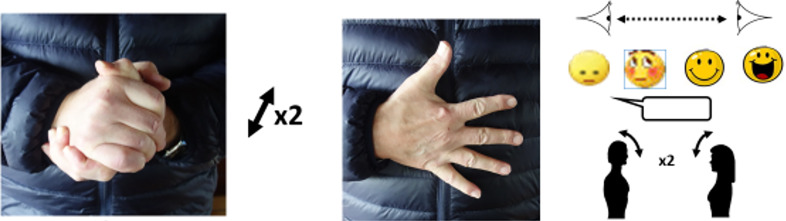
Social distancing: suggested gestures to replace close contact: “the long hand.”^20^ In the context of social distancing, Leclercq (2020)^20^ has suggested gestures that could replace close forms of contact such as hugging or kissing to communicate deep sympathy in highly emotional situations like funerals, weddings, anniversaries, and childbirth. The author advises against gestures (such as footshakes, fist-bumps or elbow-bumps) that require approaching the other person. Similarly, he rejects gestures that bear a commonly shared religious connotation (Muslim, Hindu or Christian greetings) or that have connotations of ordering, praying, begging, obeying, etc. To take advantage of the automaticity of “shaking” (in French “serrer la main = to tighten), this author recommends two gestures visible from a distance: on the left, when both hands are free, and on the right (fingers spread apart) when only one hand is free. In both cases, he recommends reinforcing these gestures by looking the addressee in the eye, uttering (audible or not, but visible) words of sympathy (as brief as possible, such as “I am with you” or the even shorter “With you”), and, finally, a small nod of the head. The signs should be customized according to the context (a sad or a happy one). These gestures were chosen for their simplicity and sensoriality (pressing hands instead of pressing the other person’s body), to avoid any similarity to religious signs or giving the impression of mimicking sign language for the deaf (which differs from country to country). Since sender and receiver should have the same understanding of such gestures, they should be promoted by mass media and social networks, so that they “go viral” like COVID-19 has. National government media outlets could get this started, after which local and private media outlets could take over and spread the message

An important part of this step is making sure that the patient develops “*an accurate mental model of the process of transmission that provides a strong rationale for what they need to do to prevent it*”.^15^ Rather than just telling people what not to do, the main goal of Step 2 is to give patients clear instructions about what they should do and why. An example (Figure 2) will illustrate the point.

At a minimum, patients should be instructed on how to take care of themselves; in that regard, see the Michie et al (2020)^6^ review of advice from the World Health Organisation, US Centers for Disease Control and Prevention, and Public Health England, setting out 13 behaviours important for reducing transmission (see Table 2). As patients’ families are usually not allowed in the triage room, patients should also be instructed on how to protect their relatives from transmission.

These recommendations should be described, demonstrated, commented upon, and practiced (at least mentally), so that patients develop a sense of self-efficacy,^21^ that is, self-esteem regarding their own capacity to perform these acts at the appropriate time, place, and frequency. This sense of mastery (what Bandura calls “self-efficacy,” or the feeling of being competent) is one of the three most important factors explaining involvement and perseverance in tasks (at least in the educational context). The other two factors^22^ are perceived value (of the actions, ie, how effective they are, and their ethical value) and perceived control (ie, does the result depend on my efforts; how much control do I have?). The latter is related to the concept of causal attribution, as described by Rotter (1990),^23^ while the former distinguishes internal locus of control (results depend upon me) from external locus of control (chance, or other factors beyond my power). Weiner (1985)^24^ distinguishes belief in the changeability or immutability of causes. The more a task is perceived as internally controllable and modifiable by the patient himself, the more likely his involvement.

As an example, consider Michie et al^6^ behaviour #9 (out of 13) : *“social distancing: if not caring for a symptomatic person, avoid contact and proximity. Maintain distance between yourself and other people, particularly those who are coughing, sneezing, or have a fever.”* The caregiver should not just give the patient models of behaviour (see “the long hand” above), but also ensure that the patient is – and feels – able and willing to perform them. Without this, there is a risk that the patient will feel powerlessness, what Seligman (1972)^25^ calls “learned helplessness” and even give up on doing those behaviours.

Clear verbal communication strategies (see Table 3) should be used to help patients better understand health information.^26^–^29^

#### Step 3 – Refer the patient to reliable resources

As the conditions for education are suboptimal (crowded facilities and very stressed patients who may be in pain), other forms of education such as written material or videos are a useful accompaniment to verbal instruction. Health professionals should steer patients to reliable websites and free helplines to prevent them from being bombarded with misinformation. All recommended resources should give evidence-based information and be easy to understand. The CDC and COVID-19 Health Literacy Project websites (www.cdc.gov/COVID19 and https://covid19healthliteracyproject.com, respectively) offer an excellent selection of such resources (see Table 4 for patient education resources).

Written instructions can be effective, provided they are not used alone and meet some basic requirements such as simplified language, large font, and a user-friendly format.^30^ However, studies that have examined the content of written instructions have found that they require an inappropriately high reading level.^31^,^32^ Written text should follow the recommendations by Flesch (1940),^33^ namely, to use short sentences and short terms (commonly used words are usually short). There is software that automatically generates Flesch Reading Ease (FRE) scores for readability (the scores range from 0–100 in English; the range varies for other languages). Readability can be tested here: https://readabilityformulas.com/free-readability-formula-tests.php. For instance, the poster named “10 things you can do to manage your covid-19 symptoms at home” (see Appendix) has a FRE score of 64.8, which can be interpreted as “Easily understood by 13–15-year-old students.”

Whenever possible, use iconic messages, since as Paivio (1968)^34^ has described, when we see a known object (or its image), its name (if known) goes automatically into working memory. He called this mechanism “dual coding.” There are various ways to test the understandability of an icon (for instance the 10 icons in the “10 Things” document). One of them consists in presenting the picture (without the text, but mentioning the theme, the title of the poster) to a sample of persons representing the target population. The testees are invited to translate the picture into words. The more the icon is translated in the same way as the (non-visible) text, the more appropriate the icon to “double” the text.

Repeat iconic messages using verbal ones (words). Shannon and Weaver (1949)^35^ demonstrated the importance of repetition in ensuring complete transmission of the information contained in a message and compensating for noise that can contradict, hamper, or – even worse – distort the intended meaning. Comment on the pictures using words and arrows. Arrows and/or crosses guide the sense of the reading; the sequence of gestures helps the brain make links, steps, and inferences. Salomon (1972)^36^ coined the term “supplantation” to describe the mechanism by which media takes charge of a mental operation rather than letting the learner’s brain conduct it itself. Some examples of how this can reduce a learner’s mental workload are the camera zooming in and out in a film, or the use of arrows in a figure to guide the reader’s gaze, or sound prompts or cues to indicate that it is time to execute an action, or heart rate devices that confirm cardiac arrhythmias that patients could/should detect themselves. Supplantation is a short-term strategy, since the patient does not learn to do these things unaided.

#### Step 4 – Explain to the patient what to do In case of emergency

Step 4 aims to enhance patients’ ability to monitor and detect symptoms that indicate worsening of their disease. To do that, health professionals tell patients about red flags (for instance, difficulty breathing, new or persistent pain or pressure in the chest, new confusion or inability to wake up, bluish lips or face, discomfort, temperature over 39°C, and headache, etc.) that should prompt them to seek medical attention or advice. Vashi and Rhodes (2011)^37^ found that although 76% of patients were given an explanation of their symptoms, only 34% were given instructions on what to do if their symptoms worsened. Therefore, it is important that health professionals prompt their patients to seek medical attention and to consult their healthcare provider for any other serious or worrying symptoms.

#### Step 5 – Check the patient’s comprehension of the information given and explore the patient’s questions

The final step involves assessing how well the patient understands the instructions. Studies in ED settings found that even when they reported high levels of satisfaction with communications, a majority of patients did not understand their diagnosis or instructions for returning.^11^ *“The literature suggests asking if patients understand is suboptimal.”*^38^ Patient discharge could be improved by two simple guidelines: use the teach-back method, and explore the patient’s questions. The teach-back method is a communication method that tests whether a patient understands what has been explained. Patients who understand are able to “teach-back” the information accurately using their own words.^39^ Systematic reviews have shown that the teach-back method yields better outcomes regarding disease-specific knowledge and better adherence to self-management instructions in chronic disease and emergency settings.^40^,^41^

Step 5 also is a key time to consider whether the patient is able to transfer what he has learned. Education is successful when the participant applies what he has learned to his behaviour. Transfer should to be assessed by asking the patient how he will apply what he has learned about prevention at home. To make at-home application of recommendations more likely, it could be suggested that visual aids be made permanent in the user’s environment (for instance by posting recommendations on the refrigerator).

Finally, a moment should be taken at the end to listen carefully to the patient’s main concerns. Healthcare providers should give patient s an opportunity to ask questions. Because it is essential that patients understand their instructions, this last step is crucial. It is natural and expected that what the learner knew before being given instructions interacts with the new knowledge, leading to new questions. Healthcare providers should give patients time to absorb the information, ask questions, and react.^42^

## DISCUSSION

Clinicians engaged in COVID-19 triage face a major challenge: that of quickly establishing an effective rapport with patients who are instructed to return home, in order to optimise patient self-management after discharge. In this context, the CEdRIC strategy can be viewed as an attempt to achieve essential goals: enabling patients to understand their medical situation; preventing complications; supporting patients; helping patients make effective use of available health services; and managing patients’ stress regarding the situation. Those goals are aligned with the core competencies described in the World Health Organisation report on patient education.^43^ Due to the acute, infectious nature of this disease, educators have to teach patients new skills such as communicating prevention measures to their families and adhering to strict self-isolation and hygiene measures to avoid transmitting the disease.

The triage context requires a new patient education format adapted to the emergency situation. First, while the recommendations generally advise allowing sufficient time for patient education and listening to what the patient knows and needs, and adapting education activities to the patient’s psychological readiness,^44^ the pandemic nature of COVID-19 demands a short format appropriate for triage and testing settings. Second, patient education in this context is by necessity less personalised and more focused on public health, with activities focused mainly on the self-isolation and hygiene measures appropriate to each patient’s situation. Third, as a consequence of the previous point, the basic steps of patient education no longer apply. In particular, the CEdRIC process bypasses two of those steps: exploring the patient’s overall needs (it focuses on knowledge rather than psychosocial needs), and negotiating the educational objectives (since the intervention is not person-dependent). It does, however, allow time for discussion at the end of the process. It takes around 15 minutes to implement CEdRIC strategy. It’s important to stick to the five steps and their related contents in order not to drift away from the main objectives of CEdRIC. Table 1 should be used as a checklist in that view.

Despite these limitations, triage, remote consultations, and discharge offer unique opportunities for teaching patients which strategies they should use to take care of themselves and limit disease transmission. While the literature offers a variety of discharge education approaches, several studies have shown that oral communication and instructional tools are relatively fast and effective techniques, and are appropriate for improving knowledge and comprehension.^30^ Effective education should incorporate health literacy concepts.^45^ This means that all of the relevant information should be delivered in a format that patients can understand.^11^ The CEdRIC strategy borrows a number of tools from the Health Literacy Universal Precautions Toolkit^26^: raising awareness; communicating clearly; using the teach-back method; and encouraging questions.

Even if the pandemic ebbs, vigilance and prevention will be needed for a long time. Health promotion actors should take the CEdRIC strategy beyond the hospital context and into the daily environments of individual citizens.

The CEdRIC strategy is currently being tested in a triage setting at the University Hospital in Liège. Since it is a health innovation, it needs to be adopted and adapted by healthcare providers. The strategy’s effectiveness must be documented as well. The COVID-19 pandemic is causing worldwide disruption. We believe that the CEdRIC strategy could be a part of the innovation so necessary to overcoming this crisis.

## CONCLUSION

Prompt detection and effective triage and isolation of potentially infected and infectious patients are a cornerstone of the pandemic response.

Discharge from triage is an opportunity to educate patients who are being instructed to return home in self-management strategies, which are the only measures currently recommended for prevention of COVID-19 transmission.

The COVID-19 pandemic requires clinicians to quickly teach their patients self-management strategies while managing the inherent pressures of an emergency situation.

The CEdRIC strategy is a practical, straightforward five-step process for delivering effective triage discharge instructions to suspected COVID-19 patients told to stay home.

The main goals of the CEdRIC approach are to provide self-management strategies for preventing complications and disease transmission.

Further study is needed to assess the CEdRIC strategy’s effectiveness.

## Supplementary Information



## Figures and Tables

**Table 1:** CEdRIC strategy: a five-step process to improve education of suspected or confirmed COVID-19 patients who are instructed to self-isolate at home.

	Steps	Objective(s)	Features/strategies	Sample sentences to be used with the patient
C	Comprehension of the situation	To inform the patient about their situation To address patient’s anxiety	Strike a balance between the seriousness of the situation and reassurance. Inform the patient about strategies for avoiding disease exacerbation and transmission.	“You are showing the symptoms of COVID-19. We can’t test you because there are not enough tests available. They are reserved for people requiring hospitalization. Hearing this makes you worried/anxious! Most patients experience mild to moderate flu symptoms (fever > 38°C, cough, headache, etc.), which take time (at least 2 weeks) to diminish or disappear. At that point they have recovered from COVID, but may still be contagious. In all of these cases (and most likely yours), you do not need to be hospitalized. There is no specific treatment for COVID-19. You must take the necessary preventive measures for yourself (to avoid secondary infection) and for others (to avoid infecting them). We can relieve your symptoms, however (antipyretic, antitussive, inhaler, etc.). We will tell you what to do.“
Ed	Patient Education about self-management strategies	To instruct the patient on how to take care of themselves and how to protect relatives from infection	Give patients clear instructions about what to do. Reinforce the patient’s sense of control, value, and self-efficacy regarding self-management strategies. Use clear verbal communication.	Stay home Monitor your symptoms carefully. Rest and drink lots of fluids. If you have a medical appointment, call the healthcare provider ahead of time and tell him or her that you have, or may have, COVID-19. Cover your cough and sneeze. Wear a face mask whenever you are around any other people. Wash your hands often. Whenever possible, stay in a specific room and away from other people in your home. Do not share your personal items with others. Clean all frequently touched surfaces.
R	References to reliable resources	To point patients to reliable websites and free helplines	Choose evidence-based, easy-to-understand references.	Resources (fill in as appropriate) (Examples from the New York State Department of Health. https://www.albanyny.gov/Government/MayorsOffice/COVID19ResourceGuide.aspx)) www.cdc.gov www.who.int You should call New York State Department of Health at 1-888-364-3065 or Albany County Department of Health at (518) 447-4580 to receive guidance on what to do and how to self-quarantine. Provision of resource materials to patients
I	Explanation about what to do in case of emergency	To bolster patients’ ability to monitor and detect symptoms of worsening disease	Inform patients about red flags that should prompt them or other family members to seek medical attention.	“Emergency warning signs include difficulty breathing; new or persistent pain or pressure in the chest; new confusion or inability to wake up; bluish lips or face; discomfort. This list may not describe all possible symptoms. Please consult your healthcare provider for any other serious or worrying symptoms”.
C	Checking the patient’s comprehension	To assess how well patients understand the instructions To make patients aware about contact tracing	Use the teach-back method Address learning transfer Give patients an opportunity to ask questions.	“We’ve talked a lot today and I want to make sure I’ve explained things properly. So let’s review what we’ve been talking about. Can you describe the main instructions on how to prevent complications and the spread of COVID-19?” (If this reveals a misunderstanding, explain again using a different approach). “What are your questions?” (Don’t say “Do you have any questions?” since most patients will respond to this by saying “no”). “You will be contacted or invited by authorities shortly to let them know your contacts during the last 7–10 days. Please cooperate actively for contact tracing in order to avoid the spread of the disease.”

**Table 2:** Thirteen behaviours to reduce transmission^6^ (© 2020 Susan Michie & BMJ Publishing Group Ltd. All rights reserved. Reproduced with permission).

Group of behaviors	Behaviors
Hand hygiene	1. Wash hands regularly with soap and water for at least 20 seconds. 2. Always wash hands: after coughing and sneezing after touching nose or mouth after caring for the sick before, during, and after food preparation before eating after using the toilet after handling animals or animal waste. 3. If soap and water are not available, use an alcohol-based hand sanitiser. This is particularly important after taking public transport.
Surface hygiene	4. Clean and disinfect frequently touched objects and surfaces in the home and work environment.
Respiratory	5. Cough or sneeze into crook of elbow or tissue. Stifle sneeze as much as possible. 6. Immediately dispose of tissue into closed bin after coughing or sneezing.
Touching	7. Do not touch mouth, eyes, or nose with unwashed hands.
Self-isolation	8. If symptomatic or otherwise advised to, stay at home for 14 days.
Social distancing	9. If not caring for a symptomatic person, avoid contact and proximity. Maintain distance between yourself and other people, particularly those who are coughing, sneezing, or have a fever.
Healthcare	10. If experiencing a fever, cough, and difficulty breathing seek medical advice early and describe previous travel history to the healthcare professional. 11. If recently arrived from specified countries within the last 14 days, call a telephone helpline.
Personal protective equipment	12. If caring for someone who has been diagnosed, wear facemasks, eye protection, and gloves.
Food safety	13. Avoid eating raw or undercooked animal products. Handle raw meat, milk, or animal organs in such a way as to avoid cross-contamination with other foods.

**Table 3:** Communication strategies to help your patients better understand health information.

Use plain, non-medical language. Speak clearly and at a moderate pace. Prioritise what needs to be discussed. Limit information to 3–5 key points. Repeat them. Duplicate verbal information with iconic messages to ensure dual coding. Reinforce verbal instructions with a written version, and follow the written version when speaking. (It can serve as a cheat-sheet.) Cite online links. Suggest where to display the written instructions at home. Give the patient the document.

**Table 4:** Coronavirus 2019 resources for patients (from the CDC^*^ and the COVID-19 Health Literacy Project).

CDC	COVID-19 Health Literacy Project
Use plain, non-medical language. Speak clearly and at a moderate pace. Prioritise what needs to be discussed. Limit information to 3–5 key points. Repeat them. Duplicate verbal information with iconic messages to ensure dual coding. Reinforce verbal instructions with a written version, and follow the written version when speaking. (It can serve as a cheat-sheet.) Cite online links. Suggest where to display the written instructions at home. Give the patient the document.	COVID-19 Prevention: This fact sheet explains how you can help prevent the spread of COVID-19. About COVID-19: This fact sheet explains what you need to know about COVID-19. Managing COVID-19: This fact sheet explains what to do if you are sick with COVID-19, or suspect you are infected. COVID-19 and pregnancy: This fact sheet explains how COVID-19 affects you if you are pregnant, or planning to become pregnant. COVID-19 for 3–6 year olds: This fact sheet can help 3–6 year olds understand the important information about COVID-19. COVID-19 for 6–12 year olds: This fact sheet can help 6–12 year olds understand the important information about COVID-19. COVID-19 for 13–18 year olds: This fact sheet can help 13–18 year olds understand the important information about COVID-19.

^*^CDC, US Centers for Disease Prevention and Control; *COVID-19*, coronavirus disease 2019.
