# Supplementary figures and images for: CEdRIC: Strategy for Patient Education During COVID-19 Triage
Source: West J Emerg Med. 2020 Oct 6;21(6):52–60. doi: 10.5811/westjem.2020.7.47907 (PMC7673883; doi:10.5811/westjem.2020.7.47907)

**Appendix.** Educational material from USA CDC ([www.cdc.gov/COVID19](http://www.cdc.gov/COVID19)).


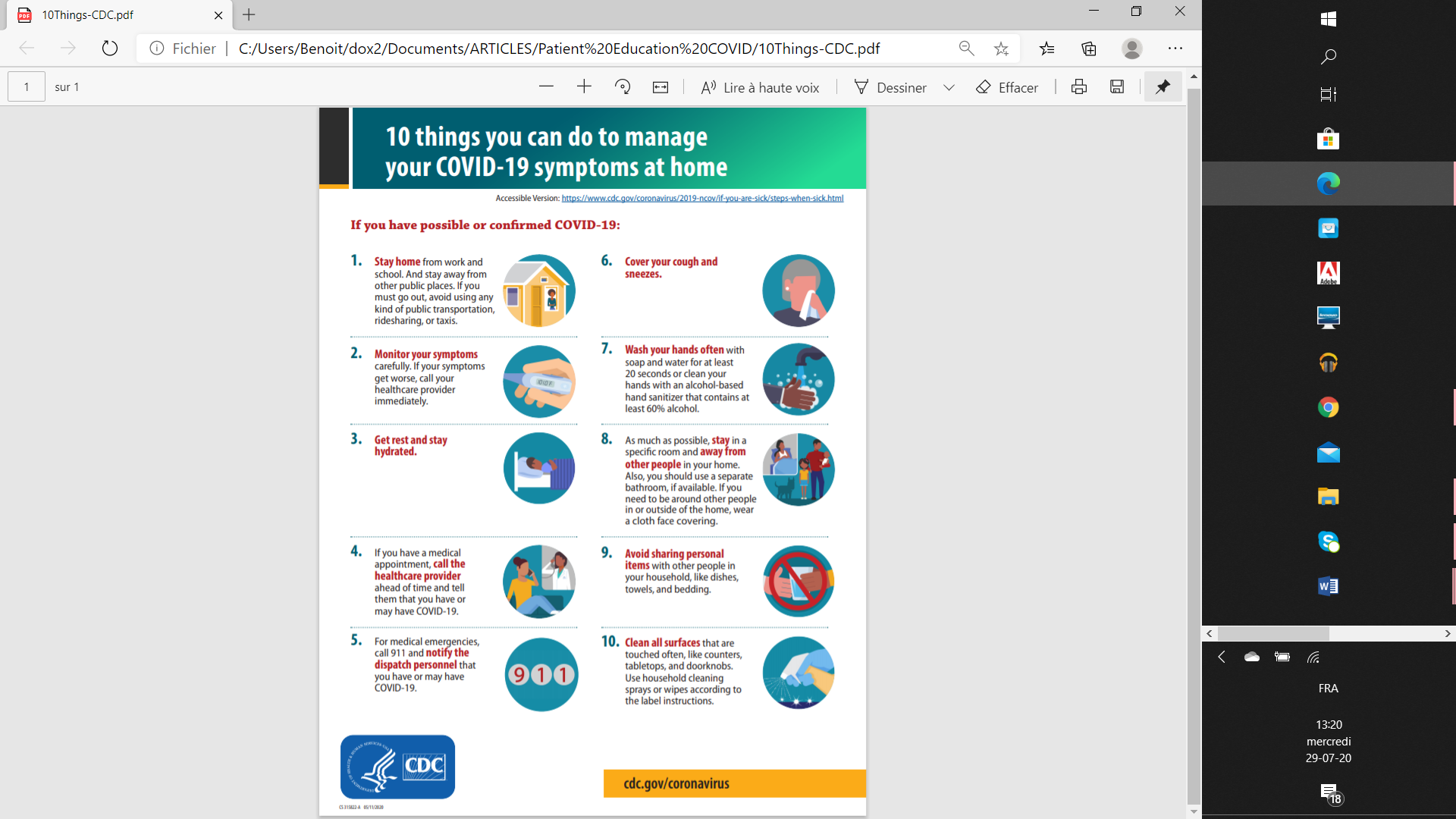

Supplement: Supplementary file 1 [file wjem-21-52-s001.docx]
